# Reply to “The Underestimation of Global Microbial Diversity”

**DOI:** 10.1128/mBio.01623-16

**Published:** 2016-09-27

**Authors:** Rudolf Amann, Ramon Rossello-Mora

**Affiliations:** ^a^ Max Planck Institute for Marine Microbiology, Bremen, Germany; ^b^ IMEDEA (CSIC-UIB), Esporles, Spain

## REPLY

Our commentary “After All, Only Millions?” (1) highlighted that, in contrast to a recent scaling law-based estimate of global microbial diversity of a trillion species-level operational taxonomic units (2), the decreasing frequencies of novel 16S rRNA gene sequences recovered by high-throughput sequencing (3, 4) is suggesting that global microbial diversity might well be saturated at a few million. No one knows even the order of magnitude of *Bacteria* and *Archaea* species numbers that ongoing surveys will ultimately yield. Acknowledging that such a census can never be truly completed, it will make a difference for many microbiologists whether we assume for our next experiments that, in addition to each species of higher plants and animals, we also need to consider either just a few hundred or a million unique microbial species in each microbiome.

We are well aware that the short-read 16S rRNA sequence repositories already contain around 1 × 10e7 different sequences, many of those only recorded once or twice. Rather than arguing that a significant part of these short sequences may be erroneous, we would like to reiterate that an accurate census should be based on long (nearly complete), high-quality 16S rRNA sequences (3, 4). Furthermore, the simple identity-based thresholds of operational taxonomic units do not record monophyly, which is a fundamental basis for the species definition of *Bacteria* and *Archaea*.

We certainly agree with our colleagues Jay Lennon and Kenneth Locey (5) that the census should include more ecosystems and that the number of candidate phyla is high (3), reflecting the deep divergence and long evolutionary history of *Bacteria* and *Archaea*. Yet, we disagree with their prediction of the extent of the rare biosphere. Our contrasting hypotheses will be brought to a test in a future in which novel sequencing technologies will provide long, error-free, and nonchimeric sequences. And even then, the debate on the global number of species of *Bacteria* and *Archaea* will continue since this relevant question cannot be resolved solely based on comparative 16S rRNA sequence analysis.

## References

[1] Amann R, Rosselló-Móra R 2016 After all, only millions? mBio 7:e01623-16. doi:10.1128/mBio.00999-16. 27381294 PMC4958260

[2] Locey KJ, Lennon JT 2016 Scaling laws predict global microbial diversity. Proc Natl Acad Sci U S A 113:5970–5975. doi:10.1073/pnas.1521291113. 27140646 PMC4889364

[3] Yarza P, Yilmaz P, Pruesse E, Glöckner FO, Ludwig W, Schleifer KH, Whitman WB, Euzéby J, Amann R, Rosselló-Móra R 2014 Uniting the classification of cultured and uncultured bacteria and archaea using 16S rRNA gene sequencing. Nat Rev Microbiol 12:635–645. doi:10.1038/nrmicro3330. 25118885

[4] Schloss PD, Girard RA, Martin T, Edwards J, Thrash JC 2016 Status of the archaeal and bacterial census: an update. mBio 7:e01623-16. doi:10.1128/mBio.00201-16. 27190214 PMC4895100

[5] Lennon JT, Locey KJ 2016 The underestimation of global microbial diversity. mBio 7:e01623-16. doi:10.1128/mBio.01298-16. 27677794 PMC5050337

